# High expression of SLC7A1 in high‐grade serous ovarian cancer promotes tumor progression and is involved in MAPK/ERK pathway and EMT

**DOI:** 10.1002/cam4.7217

**Published:** 2024-05-16

**Authors:** Shijing You, Xiahui Han, Yuance Xu, Lei Sui, Kejuan Song, Qin Yao

**Affiliations:** ^1^ Department of Obstetrics and Gynecology The Affiliated Hospital of Qingdao University Qingdao Shandong China; ^2^ Department of Gynecological Oncology Affiliated Qingdao Central Hospital of Qingdao University Qingdao Shandong China

**Keywords:** CAFs, HGSOC, SLC7A1, TGF‐β

## Abstract

Our previous studies have shown that upregulation of SLC7A1 in epithelial ovarian cancer (EOC) tumor cells significantly increases cancer cell proliferation, migration, and cisplatin resistance; however, the molecular mechanism by which SLC7A1 functions in EOC remains unknown. In later studies, we found that SLC7A1 is also highly expressed in the interstitial portion of high‐grade serous ovarian cancer (HGSOC), but the significance of this high expression in the interstitial remains unclear. Here, we showed the Interstitial high expression of SLC7A1 in HGSOC by immunohistochemistry. SLC7A1 enriched in cancer‐associated fibroblasts (CAFs) was upregulated by TGF‐β1. Transwell assay, scratch assay, cck8 assay and cell adhesion assay showed that SLC7A1 highly expressed in CAFs promoted tumor cells invasion, migration and metastasis in vitro. The effect of SLC7A1 on MAPK and EMT pathway proteins in ovarian cancer (OC) was verified by RNA sequencing and western blotting. Overexpression of SLC7A1 in OC is involved in MAPK/ ERK pathway and EMT. In general, in HGSOC, CAFs overexpressing SLC7A1 supported the migration and invasion of tumor cells; SLC7A1 is highly expressed in ovarian cancer and is involved in ERK phosphorylation and EMT signaling in MAPK signaling pathway. This suggests that SLC7A1 may be a potential therapeutic target for OC metastasis.

## Graphical abstract

Our manuscript studied that SLC7A1 enriched in cancer‐associated fibroblasts (CAFs) was upregulated by TGF‐β1; CAFs overexpressing SLC7A1 supported the migration and invasion of tumor cells in high‐grade serous ovarian cancer (HGSOC); SLC7A1 is highly expressed in ovarian cancer and is involved in ERK phosphorylation and EMT signaling in MAPK signaling pathway.

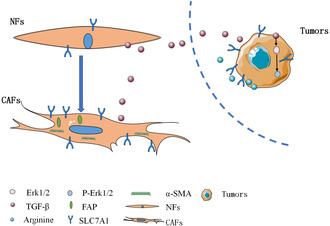

## 1 INTRODUCTION

As one of the most common malignant tumors in gynecological tumors, ovarian cancer (OC) is known for its high incidence, high mortality, high recurrence rate, and high drug resistance rate. In 2022, in the United States alone, there were 19,880 new cases of ovarian cancer and 12,810 deaths.^1^ Epithelial ovarian cancer (EOC) is the most common type of OC, accounting for about 90%,^1^ with high‐grade serous ovarian cancer (HGOSC) accounting for 70% of all EOC^2^ and 80% of all ovarian cancer deaths.^3^ At present, tumor cell reduction combined with platinum‐based chemotherapy has greatly improved the initial response rate of patients, but about 70% of HGSOC patients develop platinum‐resistant fatal disease.^4^ The malignant biological characteristics of HGSOC spread widely to the peritoneal surface in its early stage, largely depending on the communication between tumor cells and adjacent stromal cells. Among the various subtypes of HGSOC, the mesenchymal subtype has a significantly worse survival rate than the other subtypes, which is related to the increase of stromal components such as myofibroblasts and perimicrovascular cells.^5^, ^6^ Based on single‐cell sequencing analysis, tumor fibroblasts (CAFs) predominate in patients with the mesenchymal subtype of HGSOC.^7^, ^8^ Myofibroblasts and cancer‐associated fibroblasts driven by transforming growth factor‐β (TGF‐β) predicted adverse outcomes in patients with HGSOC.^9^ In HGSOC, CAFs have been proven to promote cancer cell proliferation,^10^ spread tumors through the abdominal cavity,^11^ promote immunosuppression,^12^ and mediate chemotherapy resistance. Therefore, it is of great significance to seek the target of CAFs for the treatment of HGSOC.

As a major component of the tumor matrix, CAFs are involved in signaling crosstalk between tumor cells and immune cells in the tumor microenvironment (TME).^13^ Activated CAFs specifically express α‐smooth muscle actin (α‐SMA), fibroblast activating protein (FAP), fibroblast specific protein 1 (FSP‐1), and other markers,^14^, ^15^ which promote cancer cell survival, proliferation, and invasion through increased growth factors and extracellular matrix (ECM), and enhanced metabolic activity.^16^ The contribution of CAFs to the establishment of an immunosuppressive microenvironment for ovarian cancer has also been supported by multiple evidence chains. Studies have shown that many TGF‐β signal‐related genes derived from CAFs are involved in the activation of CAFs and play an important role in the signal crosstalk between fibroblasts and ovarian cancer cells, such as COL11A1, CRMAP2, versican, POSTN, etc.^10^, ^17^, ^18^, ^19^ These proteins are involved in the continuous shuffling of the extracellular matrix, triggering a cascade of reactions and building new physical barriers that support the tumor's pathogenic processes.

SLC7A1, also known as CAT‐1, is a cationic amino acid transporter mainly involved in the transport of arginine and lysine, and is highly expressed in a variety of cancer cells to participate in tumor progression. Our previous studies have shown that SLC7A1 is highly expressed in EOC cells and is involved in tumor progression, platinum resistance, and amino acid metabolic remodeling in EOC, and is associated with poor progression‐free survival (PFS) in OC patients.^20^ Meanwhile, the database evaluation found that there was a significant positive correlation between SLC7A1 and CAFs in OC (*p* = 0.00015). However, the expression and mechanism of SLC7A1 in CAFs remain unclear. Whether CAFs with a high proportion of HGSOC specifically overexpress SLC7A1 is a question that needs further research. In this manuscript, we found that SLC7A1 is also positively expressed in the stroma portion of HGSOC tissue, so as to further investigate whether SLC7A1 in CAFs is involved in the tumor progression of HGSOC, providing a new research idea for the treatment target of HGSOC.

## 2 MATERIALS AND METHODS

### 2.1 Cell culture and induction

Human ovarian cancer cell lines SKOV3, A2780, 0VCAR3, and human normal ovarian epithelial cell line IOSE80 were obtained from Shanghai Institute of Biochemistry and Cell Biology, Chinese Academy of Sciences, and human embryo lung fibroblasts MRC‐5 were obtained from Wuhan Procell Life Science & Technology Co., Ltd. All cells were identified by STR, and the cell lines were tested for mycoplasma contamination. Use of TGF—beta 1 (PCK091 Procell, Wuhan, China) induced 48 h to get to the cells of MRC5—CAFs, hereinafter referred to as cancer‐associated fibroblasts CAFs. SKOV3, A2780, OVCAR3, and IOSE80 were placed in DMEM medium containing 10% fetal bovine serum (FBS, Gibco) and 1% double antibody. MRC‐5 and induced MRC5‐CAFs were cultured in MEM medium containing 10% fetal bovine serum (FBS, Gibco) and 1% double antibody. All cells were cultured at 37°C in 5% CO_2_ incubator (Thermo Fisher, USA).

### 2.2 Isolation and identification of primary CAF and NOF

Primary tumor‐associated fibroblast (CAF) was isolated from fresh cancer tissue of patients with high‐grade serous ovarian cancer and primary normal fibroblast (NOF) were isolated from noncancerous ovarian tissue of patients with hysterectomy and ovariectomy due to uterine fibroids. The fresh tissue was taken within 30 min and fully cleaned with ice PBS to isolate the tissue aseptically. Then they were placed in 1 mg/mL type IV collagenase (Solarbio Life Science, Beijing, China) and 0.1 mg/mL hyaluronidase, digested on a track vibrator at 37°C for 3 h, and the cell suspension was collected and centrifugated at 1200 rpm for 10 min. The collected cells were cultured in DMEM/F12 (HyClone, UT, USA) containing 15% fetal bovine serum, 1% double antibody, and 1% growth factor, and identified by α‐SMA. Informed written consent was obtained from all patients before surgery.

### 2.3 Cell transfection

The lentiviral vector (GV493) containing SLC7A1 short hairpin RNA (shRNA) was obtained by Genechem Co., Ltd. (Shanghai, China). The shRNA sequence is shown in Table S1. SKOV3, OVCAR3, MRC‐5, and CAFs cells were transfected according to manufacturer's instructions. Stable transmutation was obtained by screening with 5% puromycin after transfection. Real‐time fluorescent quantitative PCR and Western blot were used to determine the transfection efficiency.

### 2.4 Immunohistochemical staining

Approved by Ethics Committee of Qingdao University (Number: QYFY WZLL 28095), immunohistochemical staining was performed on 20 cases of high‐grade serous ovarian cancer, five normal ovarian tissue (N‐ovary), and five normal fallopian tube (N‐FT) tissue from Affiliated Hospital of Qingdao University. After paraffin embedded the tissue, the tissue was cut to 4 mm thickness, dewaxed and rehydrated, citrate solution was used for high pressure antigen repair, and the sections were soaked in 3% hydrogen peroxide solution for 15 min. After PBS was washed, the tissue range was defined on the sections by immunohistochemical pen. The sections were sealed with 10% goat serum successively for 30 min, and rabbit anti‐SLC7A1 polyclonal antibody (1: 50; Proteintech, Wuhan, China) was placed at 4°C overnight. After washing with PBS, secondary antibody (PV‐9000, Beijing, China) was incubated at room temperature for 30 min, and then diaminobenzidine (DAB) was added for color development. After color development was terminated, hematoxylin was re‐dyed and sealed.

### 2.5 Real‐time fluorescence quantitative PCR

Total RNA was extracted from cells by Trizol (TaKaRa, Japan). Then, according to the instructions of TakaRa kit, two‐step real‐time fluorescent quantitative PCR (qRT‐PCR) was used to detect the relative expression of genes. The gene expression levels of target genes relative to GAPDH were compared by calculating the threshold cycle (2^−ΔΔCT^), and the primers used were shown in Table S2.

### 2.6 Western blotting assays

Total proteins are extracted from cells or tissues using cell lysate (Cat#P0013, Beyotime Biotechnology, China). Electrophoretic protein isolation with PAGE rapid gel kit (Cat# PG111, EpiZyme Biotechnology, China) was selected according to molecular weight. The protein was then transferred to a 0.45 μm PVDF membrane (Millipore, Massachusetts, USA) by constant current electrotransfer. 5% skim milk powder was closed at room temperature for 2 h, then washed with PBS for three times, and incubated at 4°C overnight. The next day, the first antibody was recovered, the membrane was washed three times, and the second antibody was closed at room temperature for 1 h. After closure, protein bands were detected with enhanced chemiluminescence solution (Millipore, MA, USA). The dilution rates of primary and secondary antibodies for Western blot analysis are shown in Table S3. The complete blotting image was clipped, the irrelevant part was removed, and the strip image corresponding to the molecular weight was retained. The cropped image has a clear border, the cropped does not alter or distort the original data, does not affect the interpretation and quantification of the results, and does not manipulate the data in any way. The raw data with marker is uploaded to the supplementary information file.

### 2.7 Preparation of conditioned medium (CM)

The 70%–80% fused CAFs cells were simply washed twice with PBS, and the MEM medium containing 2% serum was replaced. The supernatant was collected 24 h later, and the supernatant was collected by centrifugation at 3000 rpm at 4°C for 15 min. The cell debris was removed by a 0.22 μm filter and stored at −20°C.

### 2.8 Cell proliferation assay

SKOV3 or OVCAR3 cells were inoculated on 96‐well plates. CAFs conditioned medium (CAFs‐CM) and tumor growth medium were prepared in a 2:1 ratio. The cells were cultured in the mixed medium for 0, 24, 48, and 72 h, respectively, and replaced with fresh mixed medium every 2–3 days. According to the manufacturer's instructions, the cells were added with 10 μL CCK8 solution (MedChem Express, China), incubated at 37°C for 1 h, and OD values were measured at 450 nm.

### 2.9 Invasion and migration assays

The treated CAFs cells were inoculated in the lower compartment of a 24‐well plate (Corning, NY, USA) by transwell cell coculture. The upper chamber is laid with Matrigel (Yeasen Biotechnology, Shanghai, China) in advance and hydrated. Ovarian cancer cells SKOV3 and OVCAR3 (1 × 10*5/200 μL) were inoculated in the upper chamber, incubated at 37°C for 24 h, and the unpenetrated cells and matrigel in the upper chamber were removed. The cells were then fixed with 4% paraformaldehyde and stained with 0.1% crystal violet (Solarbio, Beijing, China). Five fields of view were randomly selected under the microscope. The passing cells were photographed and counted under an optical microscope (Olympus) at 200× magnification. The experiment was repeated independently for three times. The cell migration experiment was similar to the invasion experiment, except that the substrate was not added to the upper chamber.

### 2.10 Wound healing assay

The cells were inoculated in a 6‐well plate, which was covered with a single layer overnight, and then a fixed diameter wound was marked with a 200 μL yellow gun tip, and the suspended cells were quickly washed away with PBS. The tumor growth medium DMEM: CAFs‐CM (CAFs CM for the control, shNC‐treated CAFs CM for shNC group, and shSLC7A1‐treated CAFs CM, respectively) was mixed according to the ratio of 1:2 to obtain different conditioned media. Each group of cells were incubated in different conditioned media for 48 h at 37°C in an incubator and photographed under an optical microscope. The migration area was measured by ImageJ software, the migration percentage was calculated using the initial scratch area, and the experiment was independently repeated three times.

### 2.11 Cell adhesion assay

SKOV3 cells and OVCAR3 cells were cultured in a mixture of tumor growth medium: CAFs‐CM (1:2) for 24 h, then the cells were reseeded into 96‐well plates and cultured for 30 min. The nonadherent cells were washed away by PBS, and each adherent cell was photographed under an optical microscope. Image J was used to calculate the number of cells under the visual field.

### 2.12 RNA sequencing and bioinformatics analysis

Dataset GSE40266 was used to analyze the expression levels of SLC7A1 in normal fibroblasts before and after TGF‐β1 and TGF‐β2 induction. From the cancer genome atlas (TCGA) database (https://portal.gdc.cancer.gov/) to obtain the data of 376 patients with ovarian serous carcinoma RNAseq. The gene profiles contained in the corresponding pathway were collected and analyzed through R software GAVA package. The parameter method = ‘ssgsea’ was selected, and the enrichment scores of each sample on each pathway were calculated successively according to the ssGSEA algorithm, so as to obtain the connection between the sample and the pathway. The relationship between SLC7A1, TGF‐β, and EMT pathway enrichment scores was analyzed by Spearman correlation. All of the above analysis methods and R packages were performed using v4.0.3 R software.^21^, ^22^, ^23^ *p* < 0.05 was considered statistically significant.

OVCAR3 cells were transfected with SLC7A1 shRNA or negative control shRNA. High‐throughput mRNA‐seq was conducted and the resulting data were analyzed by Biomarker Technologies (APTBIO, Shanghai, China). Appropriate cutoff values were selected, and there were significant differences in the expression of some mRNA between the two groups (*p* < 0.05 and | log_2_FC >1|). The pathway enrichment of differential genes was also performed.

### 2.13 Statistical analysis

GraphPad Prism 8.0 (GraphPad Software, Inc.) was used to analyze the data and plot them. The data were expressed as the mean ± standard deviation of at least three independent experiments. *p* < 0.05 was considered statistically significant.

## 3 RESULTS

### 3.1 SLC7A1 is highly expressed in the stroma of HGSOC

**FIGURE 1:**
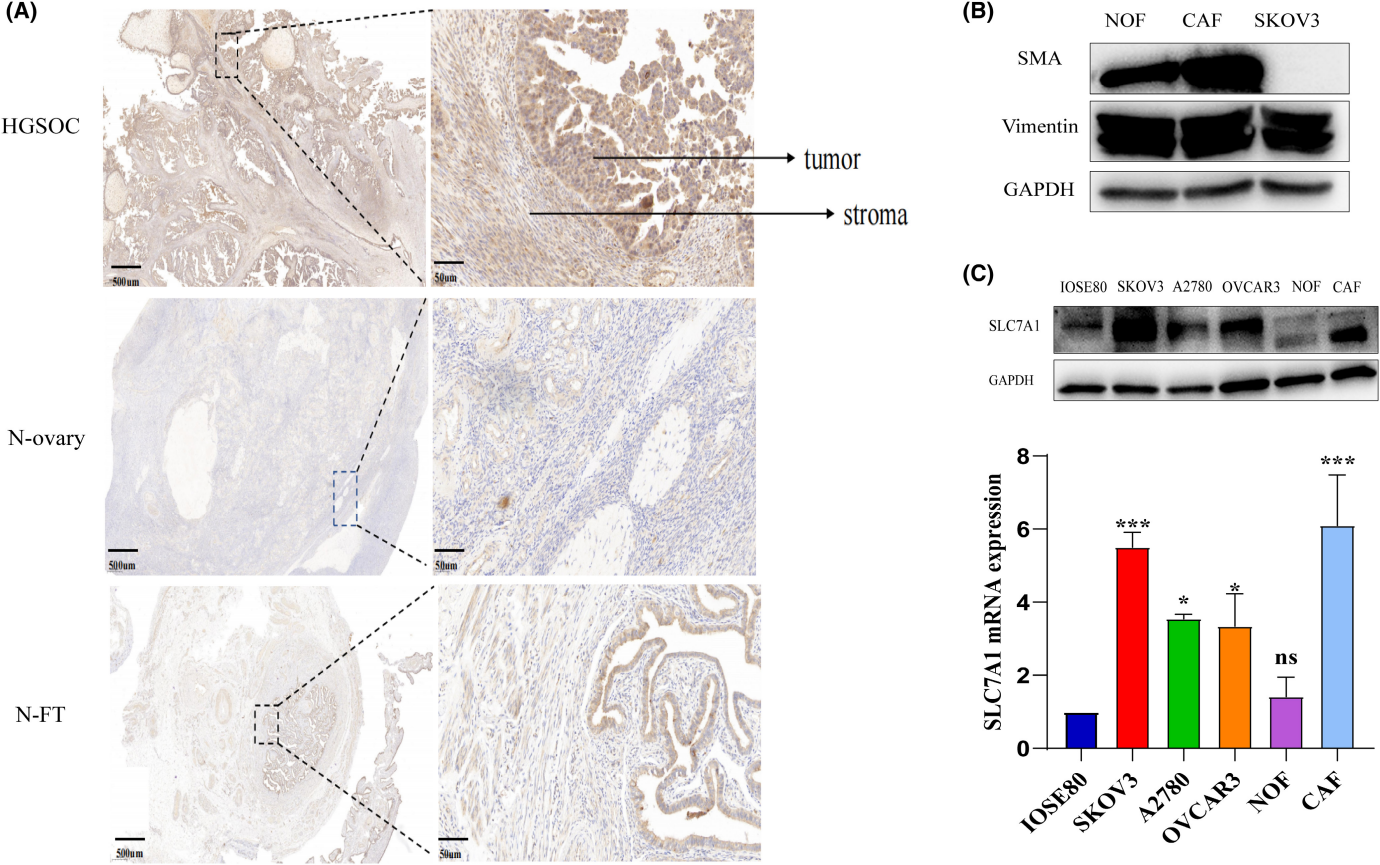
Expression of SLC7A1 in the stroma of HGSOC. (A) Immunohistochemistry showed the expression of SLC7A1 in HGSOC, normal ovary (N‐ovary) and normal fallopian tubes(N‐FT). The scales are 500 μm and 50 μm, respectively. (B) Western blotting analyzed the expression of its representative markers (α‐SMA, vimentin). (C) Western blotting and qRT‐PCR showed the expression of SLC7A1 in IOSE80, SKOV3, A2780, OVCAR3, NOF, and CAF. *p < 0.05, **p < 0.01, ***p < 0.001, *n* = 3.

Our previous bioinformatics analysis showed that overexpression of SLC7A1 was correlated with decreased progression‐free survival (PFS) in OC patients, and SLC7A1 expression in OC was closely correlated with CAFs (*p* < 0.05).^20^ To further clarify the relationship between SLC7A1 and the interstitial of ovarian cancer, immunohistochemical results showed significant SLC7A1 interstitial deposition in HGSOC samples (Figure 1A). However, perhaps due to the small number of immunohistochemical cases, we did not find significant differences in the expression of SLC7A1 in different stages of HGSOC. Primary CAF and NOF were isolated from patients with HGSOC, expressing the specific markers α‐SMA and vimentin. Compared with NOF, α‐SMA expression was significantly increased in CAF, while vimentin was not affected (Figure 1B). QRT‐PCR and Western blotting were used to detect the expression of SLC7A1. Compared with normal ovarian cells and normal fibroblasts, the expression of SLC7A1 in ovarian cancer cell lines and CAF was enhanced (Figure 1C).

### 3.2 SLC7A1 is involved in TGF‐β1‐induced fibroblast activation

**FIGURE 2:**
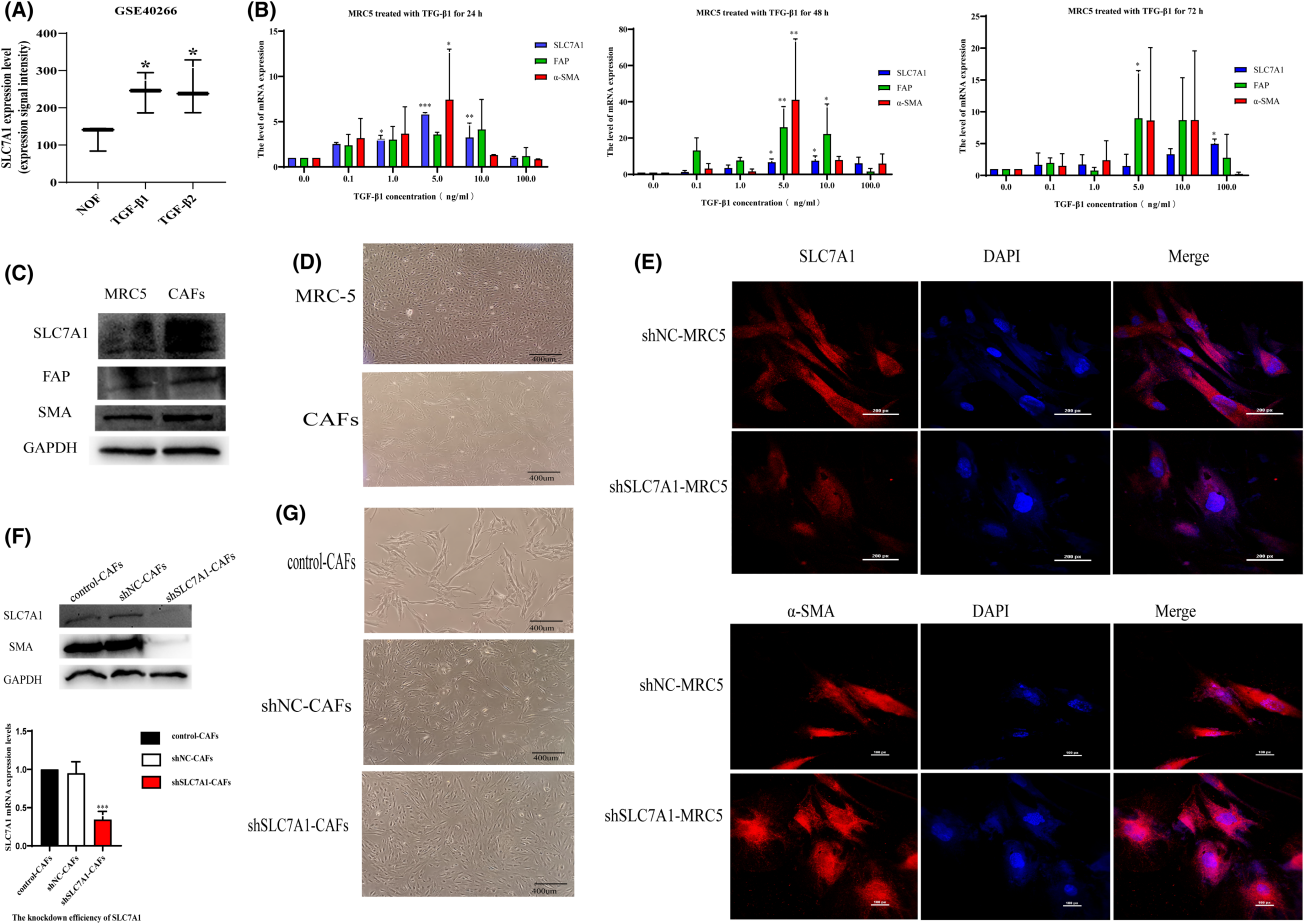
SLC7A1 is involved in TGF‐β‐mediated activation of fibroblasts (A) The GSE40266 dataset provided the expression of SLC7A1 before and after TGF‐β induction in human ovarian fibroblasts; (B) The expressions of SLC7A1, α‐SMA, and FAP in CAFs were detected by qRT‐PCR at different time and concentration of TGF‐β1. (C) Western blotting detected the expressions of SLC7A1, FAP, and α‐SMA in MRC5 cells after being induced by 5 ng/mL TGF‐β1 for 48 h. (D) The morphological changes of MRC5 cells after 48 h induced by 5 ng/mL TGF‐β1. (E) The effect of SLC7A1 knockdown on α‐SMA expression in MRC5 cells was detected by cellular immunofluorescence assay. The scales are 200px and 100px, respectively. (F) Western blotting detected the effect of SLC7A1 knockdown on α‐SMA expression in CAFs. (G) Effect of SLC7A1 knockdown on cell morphology in CAFs; *p < 0.05, **p < 0.01, ***p < 0.001, *n* = 3.

In the GSE40266 dataset, expression of SLC7A1 mRNA was enhanced in human normal ovarian fibroblasts treated with TGF‐β1 and TGF‐β2, respectively (Figure 2A). According to the Cancer Genome Atlas (TCGA), TGF‐β1 is the most commonly expressed subtype in most human cancers, compared to TGF‐β2 and TGF‐β3. MRC5 cells were treated at different concentrations of TGF‐β1 and at different times. QRT‐PCR was used to detect the expression of SLC7A1 and CAF markers, and the results showed a significant positive correlation between the expression of SLC7A1, FAP, and α‐SMA (Figure 2B). Moreover, when the concentration of TGF‐β1 was 5 ng/mL, the mRNA expression of SLC7A1, FAP, and α‐SMA changed the most, and the cell morphology changed from regular long spindle to irregular polygonal cells (Figure 2C,D). These data suggest that the upregulation of SLC7A1 in CAFs is dependent on TGF‐β1. After referring to many literatures and based on the previous research of our research group,^10^, ^24^, ^25^, ^26^, ^27^, ^28^ we treated MRC5 cells with 5 ng/mL TGF‐β1 for 48 h to obtain tumor‐associated fibroblasts (CAFs), which were later referred to as CAFs. After transfecting MRC5 with SLC7A1 interfering lectin virus, it was found that knockdown of SLC7A1 in MRC5 cells significantly inhibited cell proliferation, and the cells quickly died within 1 week (Figure S1). It was observed by cellular immunofluorescence that knockdown of SLC7A1 resulted in significant changes in cell morphology and α‐SMA was diffused in knockdown cells (Figure 2E) To further evaluate whether SLC7A1 is involved in TGF‐β1‐induced activation of CAFs, knockdown SLC7A1 in CAFs, Western blotting and qRT‐PCR were used to detect knockdown efficiency, the expression of SMA was reduced, and the cell morphology was reversed to that of normal fibroblasts MRC5 (Figure 2F,G). This suggests that SLC7A1 plays an important role in TGF‐β1‐induced phenotypic transformation of CAFs.

### 3.3 Overexpression of SLC7A1 in CAFs promotes invasion and metastasis of ovarian cancer cells

**FIGURE 3:**
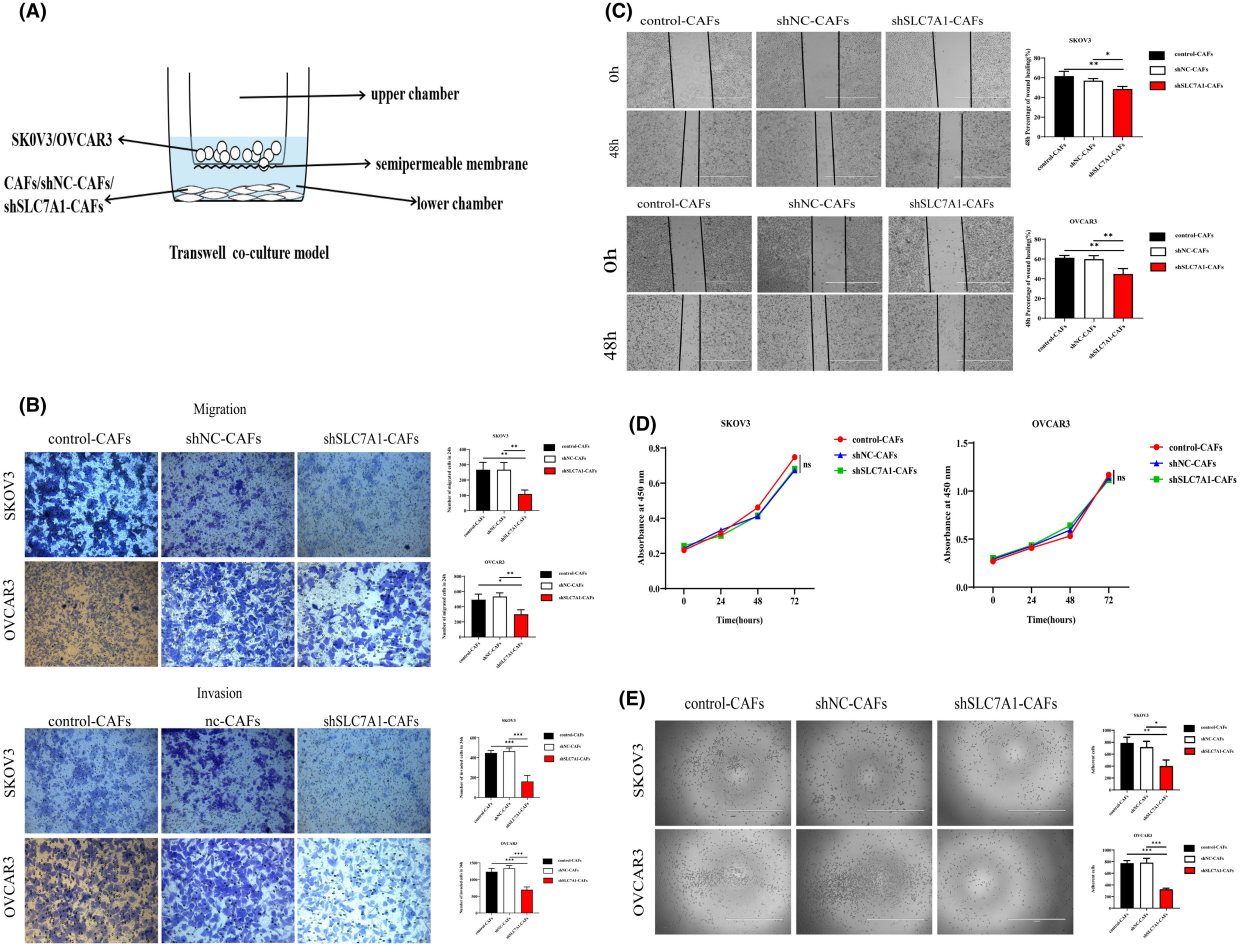
SLC7A1 knockdown in CAFs can inhibit the migration, invasion, and in vitro metastasis of OC cells. (A, B) Transwell assay was used to detect the effect of SLC7A1 knockdown on tumor cell migration and invasion in CAFs. (C) The effect of SLC7A1 knockdown on OC cell migration in CAFs was evaluated by scratch test. Scale bar = 1000 μm. (D) The effect of SLC7A1 knockdown in CAFs on the proliferation of OC cells was detected by cck8 assay. (E) OC cells were cultured with CAFs‐CM for 24 h, and then the plates were re‐laid. After 30 min, the suspended cells were washed away by PBS, and the adhesion experiment was conducted by recording the number of adherence cells. Scale bar = 1000 μm. *p < 0.05, **p < 0.01, ***p < 0.001, *n* = 3.

To investigate whether SLC7A1 is involved in the invasion and migration phenotype of TGF‐β1‐activated fibroblasts, We established a transwell coculture model, inoculated TGF‐β1‐activated CAFs in the lower compartment, and reduced the expression of SLC7A1 in CAFs. The migration and invasion of SKOV3 and OVCAR3 cells in the upper compartment of ovarian cancer were significantly reduced (Figure 3A,B). Next, the effect of SLC7A1 in CAFs on tumor cell migration was examined by scratch assay. We prepared conditioned medium (CM) for shSLC7A1‐CAFs, shNC‐CAFs, and control‐CAFs. Compared with shNC‐SLC7A1, wound healing ability of SKOV3 and OVCAR3 cells cultured with shSLC7A1‐CAFs CM for 48 h was significantly reduced (SKOV3: 57.02% vs. 48.54%, *p* < 0.05, OVCAR3: 59.85% vs. 44.79%) (Figure 3C), However, when the proliferation of cancer cells was detected by CCK8, similar proliferation rates were observed in CM cultured in the control, shNC, and shSLC7A1 groups (SKOV3: *p* = 0.0648, OVCAR3: *p* = 0.0778) (Figure 3D), indicating that the pro‐invasiveness of SLC7A1 was not related to the proliferation process.

The increased adhesion between tumor cells helps tumors migrate to new locations, thereby establishing new tumors in the body. We used cell adhesion experiments to evaluate the effect of SLC7A1 in CAFs on the ability of cancer cell metastasis (Figure 3E), Compared with shNC‐CAFs, shSLC7A1‐CAFs significantly inhibited the adhesion of EOC cells (SKOV3: 716 vs. 398.3, *p* = 0.0193, OVCAR3:781 vs. 321.3, *p* < 0.0001).

### 3.4 High expression of SLC7A1 is involved in the MAPK/ERK and EMT pathways of ovarian cancer

**FIGURE 4:**
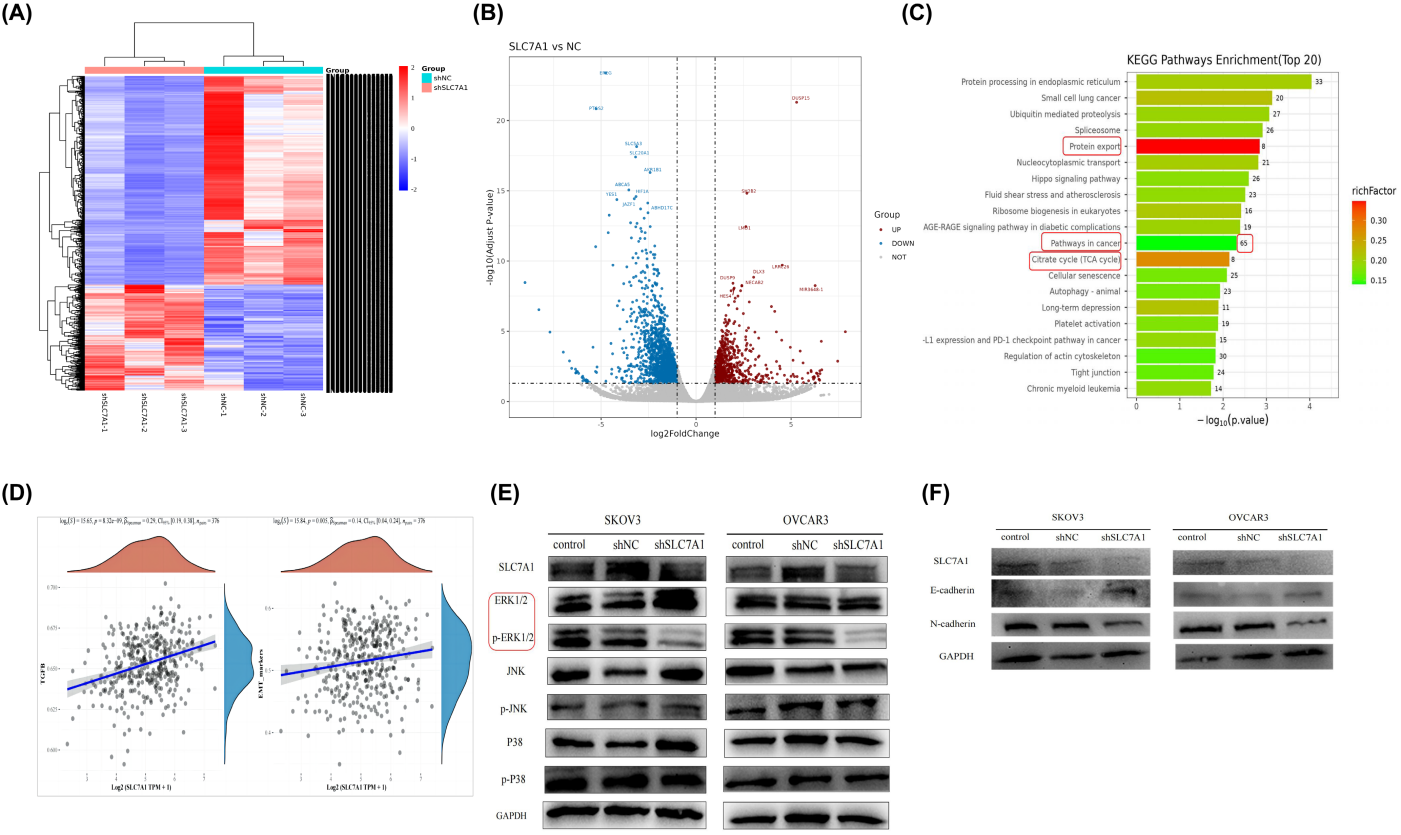
High expression of SLC7A1 in ovarian cancer is involved in MAPK and EMT pathways. (A) OVCAR3 cells were stably transfected with SLC7A1 shRNA (shSLC7A1) or negative control shNC virus. The mRNA expression profiles of shSLC7A1 and shNC groups were compared by high‐throughput RNA sequencing. (B) The volcano map showed the differentially expressed genes (DEG) between shSLC7A1 and shNC, with a total of 783 genes upregulated (up) and 1539 genes downregulated (down), and the differences were statistically significant (*p* < 0.05). (C) Kyoto Encyclopedia of Genes and Genomes (KEGG) enrichment analysis was performed for the top 20 differentially downregulated genes in shSLC7A1 group; (D) The relationship between SLC7A1 and TGFβ and EMT pathway in high‐grade serous ovarian cancer was analyzed by GSEA. (E) Western blotting detected the effects of SLC7A1 knockdown on MAPK signaling pathway protein expression in ovarian cancer cells SKOV3 and OVCAR3. (F) Western blotting detected the effects of SLC7A1 knockdown on EMT signaling pathway protein expression in ovarian cancer cells SKOV3 and OVCAR3. *p < 0.05, **p < 0.01, ***p < 0.001, *n* = 3.

To understand the mechanism by which SLC7A1 promotes malignant progression of ovarian cancer, SLC7A1 was knocked down in OVCAR3 cells, and transcriptional sequencing was used to evaluate the changes in RNA‐seq between shNC‐OVCAR3 and shSLC7A1‐OVCAR3 groups (Figure 4A,B). KEGG enrichment analysis was performed for the top 20 differentially regulated genes in shSLC7A1 group. Analysis showed that protein export, citrate cycle (TCA cycle), and pathways in cancer were highly enriched in the knockdown group (Figure 4C). Further exploring the signaling pathways in tumors, ssGSEA analysis showed that while SLC7A1 was highly expressed in patients with serous ovarian cancer, TGF‐β and EMT pathways were significantly enriched (*p* < 0.05) (Figure 4D). Since TGF‐β is involved in activating both SMAD and non‐Smad pathways, the non‐Smad pathway includes the MAPK pathway.^29^ In order to determine which of the three subgroups in MAPK signaling is responsible for the carcinogenic function of SLC7A1, Western blotting results showed that the shSLC7A1 group reduced the phosphorylation of ERK in tumor cells compared with the control group and the shNC group. Knockdown of SLC7A1 had no effect on JNK and p38 phosphorylation in tumor cells (Figure 4E). The EMT process leads to the loss of E‐cadherin in tumor cells and the high expression of N‐cadherin, which is also an important reason for first‐line chemotherapy resistance.SLC7A1 knockdown in tumor cells impaired cellular EMT progression (Figure 4F). These data suggest that SLC7A1 is involved in activating MAPK/ERK signaling and EMT in ovarian cancer cells.

## 4 DISCUSSION

Most current studies on SLC7A1 have focused on tumor cells, and our previous studies have discussed that high expression of SLC7A1 in ovarian cancer tumor cells promotes tumor malignant progression, is involved in cancer cell amino acid metabolism, and is associated with poor PFS.^20^ In this manuscript, we further found that SLC7A1 overexpression in ovarian cancer cells triggers the MAPK signaling pathway downstream of cancer cells, and expanded the significance and mechanism of SLC7A1 expression in stromal tumor‐associated fibroblasts (CAFs) of HGSOC. SLC7A1 was deposited in the HGSOC matrix, involved in TGF‐β1‐induced CAFs activation and CAFs phenotype acquisition, and promoted tumor cell progression. We also found that high expression of SLC7A1 in ovarian cancer triggers downstream MAPK and EMT pathways. Our work establishes the role of matrix SLC7A1 overexpression in fibroblast activation and promotion of HGSOC progression, and complements the possible mechanism of action of SLC7A1 in parenchymal cells. This suggests that SLC7A1 may be a promising target for HGSOC.

High‐grade serous ovarian cancer (HGSOC) is the most prevalent and aggressive subtype of EOC, accounting for more than 70% of EOCs.^30^ Gene expression studies classified HGSOC into four prognostic subtypes, of which the mesenchymal subtype was characterized by abundant stromal fibroplasia and was associated with the worst OS.^6^ Tumor‐associated fibroblasts (CAFs), as major components of the tumor matrix, have been identified as one of the drivers of tumor progression and invasion in the tumor microenvironment (TME). Among the HGSOC subtypes, overexpression of CAFs subtype (FAP^high^CD29^med‐high^SMA^high^) is associated with low survival in patients.^13^, ^31^ More and more studies have found that there is a complex signal crosstalk between CAFs and cancer cells, and CAFs in the tumor microenvironment of ovarian cancer (OC) participate in the growth and development of tumor cells by inducing tumor cell growth, proliferation, angiogenesis, and inhibiting antitumor response.^32^, ^33^ These data highlight that targeting matrix CAFs can mitigate stromal fiber development and signal crosstalk, thereby slowing disease progression. However, the molecular mechanism of CAFs in the pathogenesis of HGSOC is not very clear. In this study, we found that SLC7A1 was specifically overexpressed in the stromal portion of HGSOC, and the expression of SLC7A1 was upregulated in stromal fibroblasts subjected to TGF‐β1‐induced activation. CAFs that overexpress SLC7A1 promote invasion, migration, and cell adhesion of cancer cells (SKOV3 and OVCAR3).

SLC7A1, also known as CAT‐1, belongs to the cationic amino acid transporter family (CAT) and is a member of the solute carrier family 7 (SLC7), which is responsible for cationic amino acids (arginine, lysine, and ornithine) necessary for cells growth.^34^, ^35^ SLC7A1 plays an important role in the uptake of arginine and is involved in the regulation of T cells and tumor cells biological behavior.^36^, ^37^ In hepatocellular carcinoma (HCC), SLC7A1 specifically introduces arginine, and its inhibition slows the growth of HCC cells in vivo and in vitro.^38^ In human breast cancer cells,^39^ colorectal cancer,^35^ and ovarian cancer cells,^20^ overexpression of SLC7A1 is beneficial to the growth and survival of tumor cells, and promotes the malignant progression of tumors. In addition, the survival and differentiation of T cells cannot be achieved without the involvement of SLC7A1. L‐arginine transported by SLC7A1 enhances T cell survival and antitumor ability by inhibiting glycolysis and promoting OXPHOS and transcriptional regulators (BAZ1B, PSIP1, and TSN).^40^ However, the role of SLC7A1 in tumor stroma has not been reported. In this manuscript, transwell coculture experiments were used to find that CAFs regulated the migration and invasion ability of ovarian cancer cells through SLC7A1. As more and more tumor mutation loads are being found in CAFs, SLC7A1 is not the only gene driving ovarian cancer metastasis, as metastasis is a multisignaling, multistep complex process involving the interaction of multiple components in the tumor microenvironment. In conjunction with our previous findings, we found that SLC7A1 is highly expressed in both the parenchymal and interstitial parts of HGSOC. In both tumor cells and CAFs, the high expression of SLC7A1 promotes the malignant progression of tumors, so SLC7A1 is undoubtedly a very important gene in the progression of HGSOC.

TGF‐β plays an inhibitory role in the early stage of the disease, but in the late stage of the disease, under pathological conditions, overexpression of TGF‐β will promote epithelial interstitial transformation (EMT),^41^ extracellular matrix deposition (ECM),^42^ and the formation of cancer‐associated fibroblasts (CAFs),^43^ leading to the formation of fibrotic diseases and cancer.^44^ Activated TGF‐β stimulates downstream signaling pathways (SMAD and non‐SMAD pathways) to regulate upstream and downstream signaling transcription. In the non‐SMAD signaling pathway, mature TGF‐β activates the mitogen‐activated protein kinase (MAPK) pathway, extracellular signal‐regulated kinase 1/2 (Erk1/2) pathway, RHO‐like signaling pathway, phosphatidylinositol‐3 kinase (PI3K)/AKT pathway, c‐Jun amino‐terminal kinase (JNK), and p38 mitogen‐activated protein kinase (p38/MAPK) signaling pathway.^45^ The Erk signaling pathway influences embryonic development, especially nerve development, and is involved in EMT to promote fibrosis and cancer metastasis in aging diseases.^46^, ^47^, ^48^, ^49^ More and more studies have shown that in ovarian cancer stromal cells, mutations in target genes are involved in the combination of TGF‐β signaling and core pathway components (including ligands, receptors, SMAD, transcription factors, and MAPK).^17^, ^19^, ^28^, ^50^, ^51^ Our results show that SLC7A1 is involved in TGF‐β1‐induced fibrosis of CAFs. After SLC7A1 knockdown, the expression of α‐SMA, the activation marker of CAFs, and the phosphorylation of Erk protein are significantly decreased.

In our study, TGF‐β1 was used to induce MRC‐5 cells to become CAFs. Although this experimental method has been recognized in a number of studies on the mesenchyma of ovarian cancer, further preparation of primary CAFs and further research on the basis of primary CAFs are still needed. In addition, this study was limited to in vitro models, and indirect coculture experiments were insufficient to simulate signal crosstalk between CAFs and ovarian cancer cells. The role of SLC7A1 overexpressed in CAFs in the microenvironment of HGSOC, as well as the potential antitumor effects of targeting SLC7A1, will be further investigated in vivo.

In summary, according to our previous studies, SLC7A1 expression is elevated in both tumor parenchymal cells and CAFs in HSCOC. SLC7A1 overexpression in tumor cells promotes the proliferation, migration, invasion, and platinum resistance of cancer cells, and participates in amino acid metabolism remodeling of cancer cells. It was further found that SLC7A1 overexpressed in cancer cells participated in TGF‐β1/MAPK signaling pathway, promoting the phosphorylation of Erk MAPK pathway. The expression of SLC7A1 is upregulated during TGF‐β1‐mediated CAFs activation. SLC7A1 overexpression of CAFs promoted the invasion and migration of tumor parenchymal cells. In conclusion, SLC7A1 is a strong potential therapeutic target for HGSOC, but there is no clear target for SLC7A1. Considering that SLC7A1 mainly ingests arginine, most of the drugs currently available target energy depletion of arginine.^52^ It is worth noting that arginine depletion is a double‐edged sword in the tumor microenvironment, and its therapeutic effect in solid tumors, especially ovarian cancer cells, needs further research.

## AUTHOR CONTRIBUTIONS

**Shijing You:** Conceptualization (equal); investigation (equal); methodology (equal); resources (equal); software (equal); writing – original draft (equal). **Xiahui Han:** Formal analysis (equal); methodology (equal); software (equal); visualization (equal). **Yuance Xu:** Formal analysis (equal); investigation (equal); software (equal); validation (equal). **Lei Sui:** Data curation (equal); software (equal); validation (equal). **Kejuan Song:** Supervision (equal); validation (equal). **Qin Yao:** Conceptualization (equal); data curation (equal); funding acquisition (equal); validation (equal); writing – review and editing (equal).

## CONFLICT OF INTEREST STATEMENT

The authors state that the study was published without any conflict of interest.

## Supporting information


Appendix S1.

## Data Availability

The original data generated during the experiment can be reasonably obtained from the corresponding author, and some data related to bioinformatics can be found in the hyperlink of the manuscript.
