# Supplementary material for: High expression of SLC7A1 in high‐grade serous ovarian cancer promotes tumor progression and is involved in MAPK/ERK pathway and EMT
Source: Cancer Med. 2024 May 16;13(10):e7217. doi: 10.1002/cam4.7217 (PMC11097251; doi:10.1002/cam4.7217)
Supplement: Supplementary file 1 — Appendix S1. [file CAM4-13-e7217-s001.docx]

**SUPPLEMENTARY FIGURES AND TABLES**


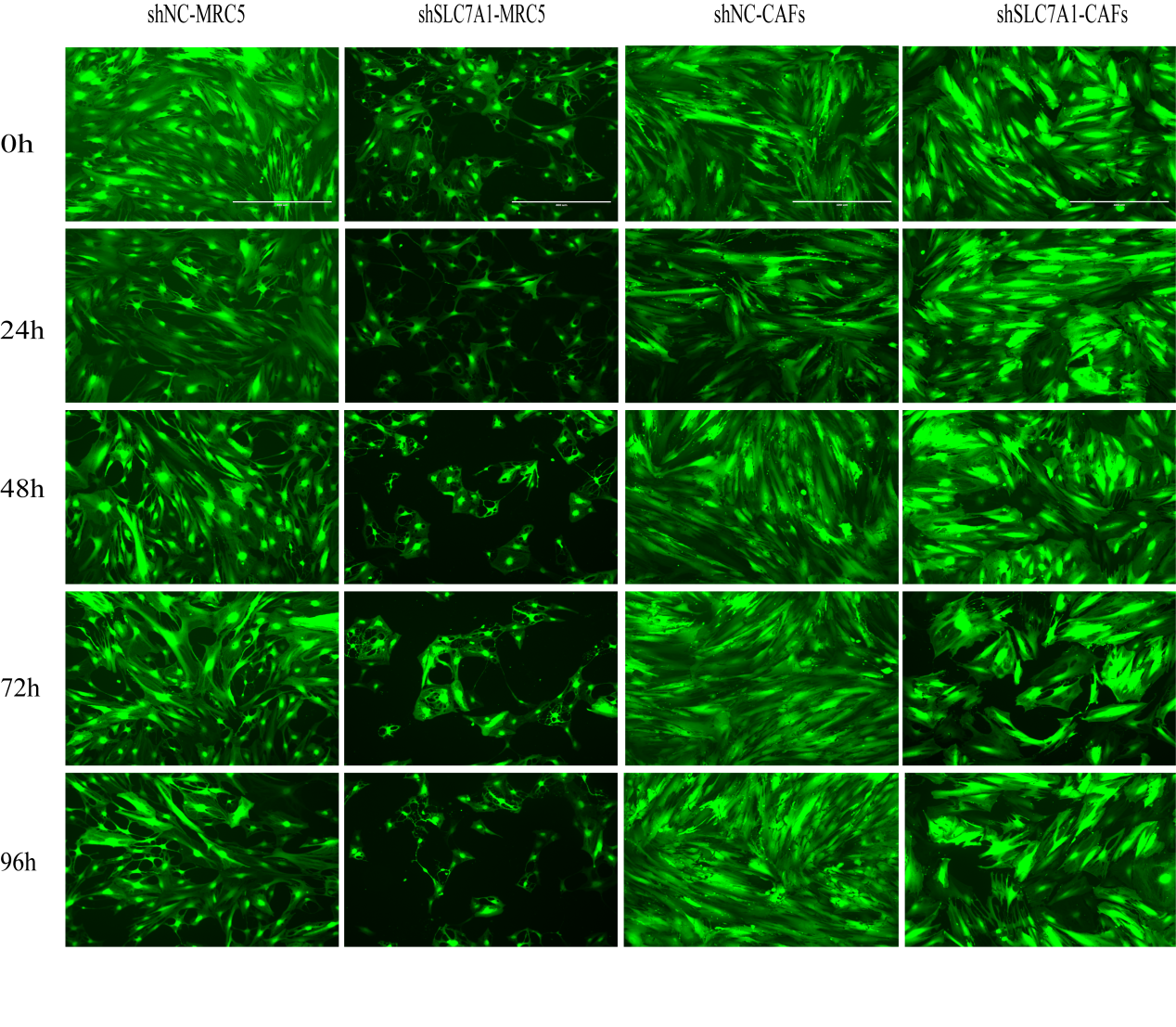


Supplementary Figure S1：After SLC7A1 was knocked down in MRC5 cells and CAFs cells, the changes of cell morphology and growth state were observed every 24 hours；scale bar=400um.

**Supplementary table S1.** Sequences of shRNA

| **shRNA Name** | **Sense** | **Antisense** |
| --- | --- | --- |
| shSLC7A1 | CCGGCTGGGCTAATTGTGAACATTTCTCGAGAAATGTTCACAATTAGCCCAGTTTTTG | AATTCAAAAACTGGGCTAATTGTGAACATTTCTCGAGAAATGTTCACAATTAGCCCAG |

**Supplementary table S2.** Primers used in real-time PCR, including the primer sequences for GAPDH、SLC7A1、FAP and α-SMA.

| **Gene name** | **Forward (5'-3')** | **Reverse (5'-3')** |
| --- | --- | --- |
| GAPDH | GGAGCGAGATCCCTCCAAAAT | GGCTGTTGTCATACTTCTCATGG |
| SLC7A1 | CACCAACTGGGACGACAT | AGGCGTACAGGGATAGCA |
| α-SMA | CTCTGGACGCACAACTGGCATC | CACGCTCAGCAGTAGTAACGAAGG |
| FAP | TATTCCATACCCAAAGGC | CTCGTTCATCAGTAACCC |

**Supplementary table S3. The antibodies and dilution rate used in this research**

| Name of Antibody | Company | Catalog Number | dilution rate |
| --- | --- | --- | --- |
| SLC7A1 | Proteintech | 14195-1-AP | 1：1000 |
| FAP | Affinity | AF0739 | 1：1000 |
| α-SMA | Elabscience | E-AB-34268 | 1：1000 |
| ERK1/2 | ABclonal | A4782 | 1：1000 |
| JNK1/2/3 | ABclonal | A4867 | 1：1000 |
| P38 MAPK | ABclonal | A4771 | 1：1000 |
| P-ERK1/2 | ABclonal | AP0972 | 1：1000 |
| P-JNK1/2/3 | ABclonal | AP0631 | 1：1000 |
| P-P38 MAPK | ABclonal | AP0057 | 1：1000 |
| E-cadherin | Cell Signaling Technology | 24E10 | 1：1000 |
| N-cadherin | Servicebio | GB111009-100 | 1：1000 |
| vimentin | Cell Signaling Technology | 46173 | 1：1000 |
| GAPDH | Elabscience | E-AB-20095 | 1：5000 |
| goat antirabbit IgG | Elabscience | E-AB-1003 | 1: 4000 |
